# Automated immature granulocyte count as a specific rule-in marker for bacteremia in the emergency department: A retrospective cohort study of 1418 patients

**DOI:** 10.1097/MD.0000000000049596

**Published:** 2026-07-10

**Authors:** Wan-Hua Yang, Yi-Ju Yang, Cheng-Pin Huang, Tzeng-Ji Chen

**Affiliations:** a Department of Laboratory Medicine, Taipei Veterans General Hospital Hsinchu Branch, Hsinchu, Taiwan, ROC; b Department of Industrial Engineering and Management, Yuan Ze University, Taoyuan, Taiwan, ROC; c Department of Medical Laboratory Science and Biotechnology, Yuanpei University of Medical Technology, Hsinchu, Taiwan, ROC; d Division of Nephrology, Department of Internal Medicine, Taipei Veterans General Hospital Hsinchu Branch, Hsinchu, Taiwan, ROC; e Department of Family Medicine, Taipei Veterans General Hospital Hsinchu Branch, Hsinchu, Taiwan, ROC; f Department of Family Medicine, Taipei Veterans General Hospital, Taipei, Taiwan, ROC; g Department of Post-Baccalaureate Medicine, National Chung Hsing University, Taichung, Taiwan, ROC.

**Keywords:** bacteremia, emergency medicine, immature granulocytes, sepsis, specificity

## Abstract

Rapid identification of bacteremia in the emergency department is critical for initiating timely antimicrobial therapy. While blood culture remains the gold standard, its diagnostic delay necessitates reliable surrogate markers. Traditional biomarkers like white blood cell (WBC) count offer high sensitivity but are often nonspecific, leading to unnecessary antibiotic use. This study evaluated whether the automated Immature Granulocyte (IG) count could serve as a specific rule-in marker for bacteremia. We conducted a retrospective cohort study analyzing 1418 adult patients who underwent simultaneous blood culture and complete blood count sampling at a regional teaching hospital in Taiwan throughout 2023. Patients were categorized into bacteremia (positive culture) and non-bacteremia groups. The diagnostic performance of IG% was compared with WBC, neutrophil-lymphocyte ratio, and qSOFA scores. Of the 1418 patients, 457 (32.2%) had confirmed bacteremia. While WBC (>12,000/μL) demonstrated high sensitivity (90.4%), its specificity was poor (21.7%). In contrast, an IG cutoff of >2.0% yielded a significantly higher specificity of 72.1% (sensitivity: 35.2%). Receiver operating characteristic curve analysis indicated that while IG had a modest overall area under the curve (0.51), its distribution in bacteremic patients was notably skewed towards higher values, supporting its utility as a confirmatory marker. Automated IG% exhibits superior specificity compared to traditional screening markers. We propose a 2-step strategy utilizing WBC for broad screening and IG% for targeted confirmation. Future prospective, multicenter studies are warranted to validate these findings and further evaluate the clinical impact of incorporating IG% into sepsis diagnostic algorithms.

## 1. Introduction

Sepsis and bacteremia represent critical emergencies requiring prompt recognition to improve survival outcomes.^[1,2]^ While blood culture remains the diagnostic reference standard, its clinical utility in the acute phase is limited by a turnaround time of 24 to 72 hours.^[3]^ Consequently, clinicians rely heavily on surrogate biomarkers to guide early decision-making.

Ideally, a biomarker should be both sensitive enough to detect infection and specific enough to minimize unnecessary antibiotic use. Traditional markers such as total WBC count and C-reactive protein (CRP) are widely used but are often criticized for their nonspecific elevation in response to trauma, stress, or sterile inflammation.^[4,5]^ While Procalcitonin (PCT) has emerged as a specific marker for bacterial sepsis, its high cost and lack of routine availability in all ED settings, particularly in regional hospitals, limit its universal application as a first-line screening tool. In contrast, hemogram parameters are universally available within minutes of ED arrival. Therefore, identifying a specific parameter hidden within the routine CBC, without incurring additional costs or turnaround time, represents a significant unmet need in resource-limited healthcare environments.

Recently, the neutrophil-lymphocyte ratio (NLR) has gained attention as a marker of physiological stress; however, its specificity for distinguishing invasive bacterial infection from general stress remains debated.^[6,7]^ Immature granulocytes (IG), representing precursors such as promyelocytes, myelocytes, and metamyelocytes, are historically associated with the left shift phenomenon during severe bacterial infection.^[8]^ Modern hematology analyzers now provide automated IG counts as part of the standard complete blood count (CBC), eliminating the inter-observer variability of manual review.^[9]^ Unlike NLR, which reflects a balance of immune responses,^[10]^ the release of IGs implies direct bone marrow stimulation, potentially offering higher specificity for invasive infection.^[11]^

In this study, we utilized a large dataset (N = 1418) to compare the diagnostic performance of automated IG% against NLR, WBC, and qSOFA. Specifically, we aimed to determine whether IG provides superior specificity for predicting bacteremia, thereby serving as a complementary, zero-cost tool to current screening methods.

## 2. Materials and methods

### 2.1. Study design and population

This retrospective cohort study (N = 1418) was conducted at the Taipei Veterans General Hospital, Hsinchu Branch. Data were extracted from the laboratory information system for all adult patients (≥18 years) presenting to the ED between January 1, 2023, and December 31, 2023. The study protocol was approved by the Institutional Review Board of Taipei Veterans General Hospital (IRB No: 2024-05-006CC). Patient informed consent was waived by the IRB due to the retrospective and anonymized nature of the data.

### 2.2. Data collection

We included patients who underwent both blood culture collection and a CBC test within a strict 24-hour interval to ensure temporal relevance. The Time Anchor was defined as the blood culture collection time. Bacteremia was defined as a positive blood culture yielding a pathogenic organism; contaminants (e.g., coagulase-negative staphylococci in a single bottle) were classified as negative.^[3]^

### 2.3. Predictor definitions

- IG%: Automated count from Sysmex XN-series analyzers. Abnormality defined as >2.0%.
- WBC: Abnormality defined as >12,000/μL.
- NLR: Calculated as absolute neutrophil count divided by lymphocyte count.
- qSOFA: Calculated based on respiratory rate, systolic blood pressure, and GCS score.^[2]^

### 2.4. Statistical analysis

Diagnostic performance metrics (Sensitivity, Specificity, PPV, NPV) were calculated. Receiver Operating Characteristic (ROC) curves were generated to compare area under the curve. A *P*-value <.05 was considered statistically significant.

## 3. Results

### 3.1. Baseline characteristics

**Figure 1.**
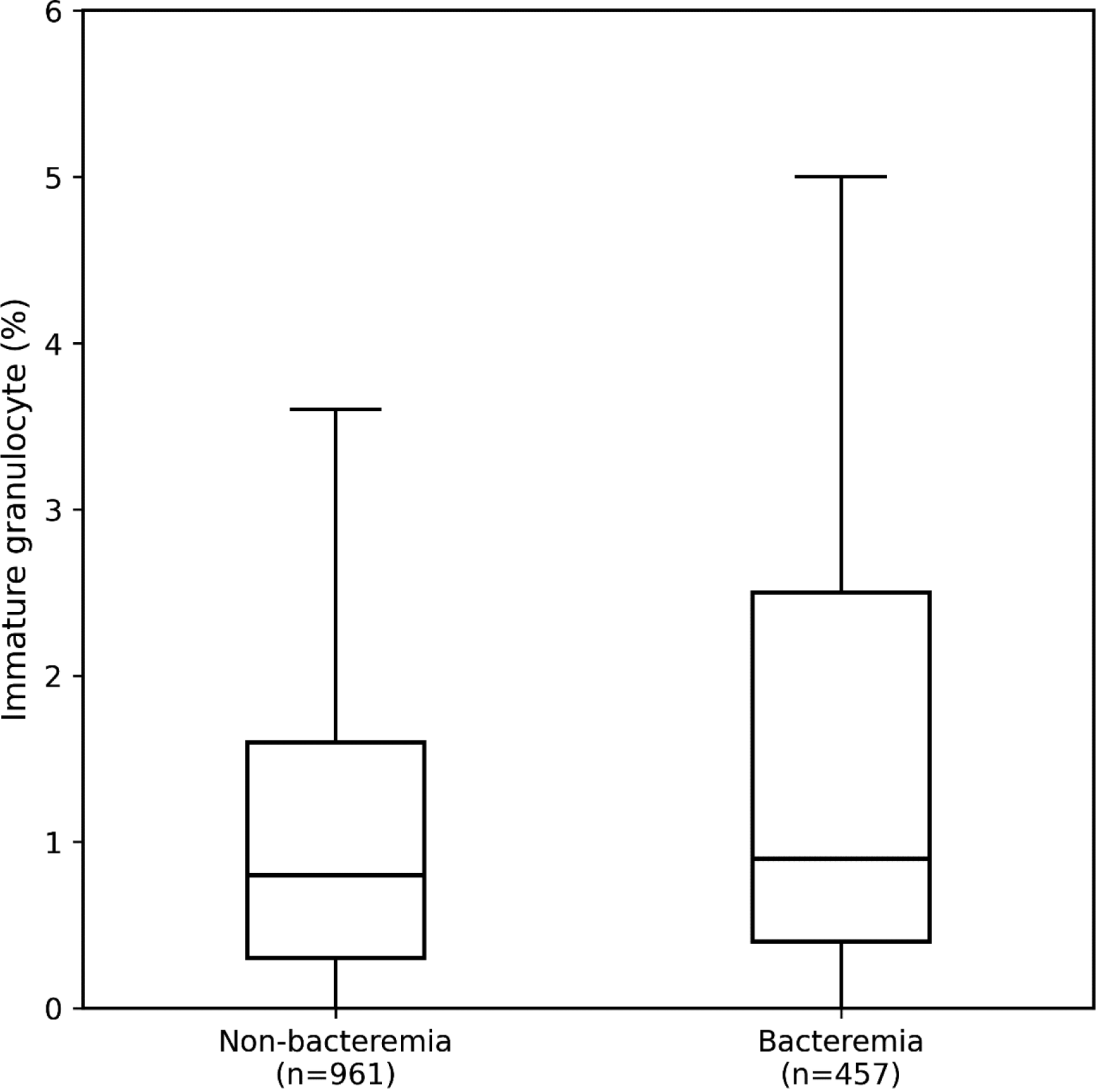
Distribution of IG% by bacteremia status. Boxplot displaying the distribution of IG percentages. The positive bacteremia group exhibits a distribution skewed towards higher values compared to the negative group. IG = immature granulocyte.

A total of 1418 patients were analyzed, with 457 (32.2%) confirmed cases of bacteremia. The bacteremia group exhibited significantly higher mean WBC counts (22.3 vs 19.4 × 10^3^/μL, *P* <.001) and a higher proportion of qSOFA scores ≥ 1 (62.8% vs 53.2%, *P* <.001) compared to the non-bacteremia group (Table 1). Despite comparable median IG% values, the distribution in the bacteremia group was skewed towards higher values (Fig. 1).

**Table 1:** Baseline characteristics of the study population (N = 1418).

Characteristic	Total (N = 1418)	Bacteremia (+) (n = 457)	Non-Bacteremia (-) (n = 961)	*P*-value
WBC (×10^3^/μL), Mean (SD)	20.3 (±8.5)	22.3 (±9.1)	19.4 (±7.8)	<.001
NLR, Median (IQR)	18.5 (9.2–32.1)	21.4 (10.5–35.6)	17.1 (8.8–30.2)	<.001
IG%, Median (IQR)	0.8 (0.4–1.8)	0.9 (0.4–2.5)	0.8 (0.3–1.6)	.08
qSOFA Score ≥ 1, n (%)	798 (56.3%)	287 (62.8%)	511 (53.2%)	<.001

Data are presented as mean (standard deviation), median (interquartile range), or number (percentage). *P*-values were calculated using the independent *t*-test, Mann–Whitney *U* test, or Chi-square test as appropriate.

IG = immature granulocyte, NLR = neutrophil-lymphocyte ratio, qSOFA = quick Sequential Organ Failure Assessment, WBC = white blood cell.

### 3.2. Diagnostic performance

As shown in Table 2, WBC (>12,000/μL) demonstrated high sensitivity (90.4%) but low specificity (21.7%). In contrast, automated IG% (>2.0%) achieved the highest specificity (72.1%) among the hematological markers, establishing it as a strong “rule-in” indicator. NLR showed moderate performance with a specificity of 64.2%.

**Table 2:** Diagnostic performance for predicting bacteremia.

Biomarker (cutoff)	Sensitivity (95% CI)	Specificity (95% CI)	PPV (%)	NPV (%)
WBC (>12,000/μL)	90.4%	21.7%	35.4	82.5
NLR (>30.3)	51.0%	64.2%	40.4	73.4
IG% (>2.0%)	35.2%	72.1%	37.5	70.1
qSOFA (≥1)	62.8%	46.8%	36.0	72.8

CI = confidence interval, IG = immature granulocyte, NLR = neutrophil-lymphocyte ratio, NPV = negative predictive value, PPV = positive predictive value, qSOFA = quick Sequential Organ Failure Assessment, WBC = white blood cell.

### 3.3. ROC curve analysis

**Figure 2.**
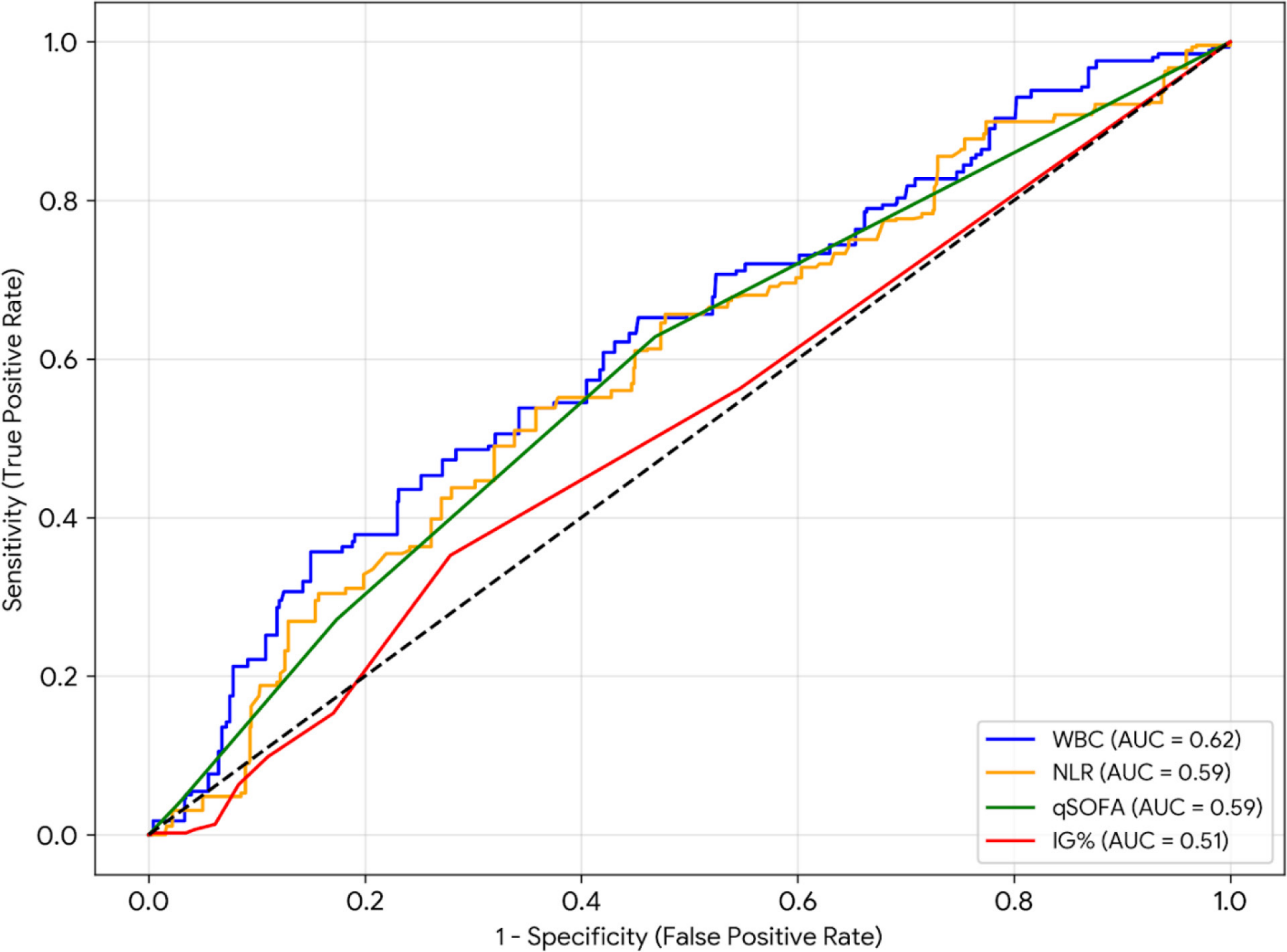
ROC curves for predicting bacteremia. Comparison of diagnostic performance among WBC (AUC = 0.62), NLR (AUC = 0.59), qSOFA (AUC = 0.59), and IG% (AUC = 0.51). Note the IG curve’s trajectory in the high-specificity region (lower left), supporting its role as a confirmatory rule-in marker. AUC = area under the curve, IG = immature granulocyte, NLR = neutrophil-lymphocyte ratio, qSOFA = quick sequential organ failure assessment, ROC = receiver operating characteristic, WBC = white blood cell.

ROC analysis (Fig. 2) revealed that while WBC achieved the highest area under the curve (0.62) due to its sensitivity, the IG% curve highlighted its distinct value in the high-specificity region, reinforcing its role as a confirmatory rather than a screening test.

## 4. Discussion

Our study clarifies the specific clinical utility of automated IG counts in diagnosing bacteremia. By comparing IG with NLR and WBC in a large cohort, we demonstrate that while IG is not a standalone screening tool, its elevated specificity makes it a valuable complementary marker.

### 4.1. Specificity advantage and pathophysiological mechanism

A major limitation of current sepsis biomarkers is nonspecificity. Markers like WBC and NLR reflect the general mobilization of the marginal pool in response to stress, trauma, or viral infections,^[12]^ leading to high sensitivity but poor specificity. Theoretically, the presence of immature granulocytes in peripheral blood indicates a profound stimulation of the bone marrow reservoir, mediated by cytokines such as G-CSF, which overrides the marrow’s retention mechanisms.^[13]^ This phenomenon implies a systemic demand for neutrophils that exceeds the steady-state supply, a condition most strongly associated with invasive bacterial infection rather than transient physiological stress. Our empirical data supports this biological plausibility: while WBC counts were easily elevated by non-bacteremic stressors, significant IG elevation (>2.0%) was far more specific (72.1%) to culture-proven bacteremia.^[14,15]^

### 4.2. Clinical application: a 2-step strategy

We propose a pragmatic integration of these markers. Clinicians should continue using WBC/NLR as initial screening filters. However, when these are elevated, checking the IG% can refine risk assessment. While the specificity of 72.1% implies that IG is not a definitive standalone diagnostic test, it is significantly superior to the 21.7% specificity of WBC. In the context of ED screening, where false alarms from WBC are prevalent, IG serves as a valuable relative rule-in marker to increase the posttest probability of bacteremia. Consequently, a concomitant rise in IG (>2%) significantly raises the suspicion of bacteremia, justifying blood culture collection in ambiguous cases.

### 4.3. Comparison with literature

Our findings regarding the high specificity of IG align with previous investigations, though with notable distinctions in diagnostic sensitivity. Nierhaus et al reported that IG counts could discriminate between SIRS and sepsis with a sensitivity of 80% and specificity of 91%.^[8]^ However, it is crucial to note that their study used clinical sepsis (Sepsis-2 or 3 criteria) as the outcome. In contrast, our study strictly defined the outcome as culture-proven bacteremia. This rigorous microbiological standard explains why our observed sensitivity (35.2%) was lower than studies focusing on broader clinical syndromes. Bacteremia is a specific subset of sepsis; therefore, while IG is less sensitive for general sepsis, our data suggests it is highly specific for the invasive presence of bacteria in the bloodstream.

Regarding NLR, our results corroborate the findings by Ljungström et al^[5]^ and Zahorec,^[6]^ which established NLR as a sensitive indicator of systemic stress. However, our data extends their findings by demonstrating that while NLR correlates well with WBC, it lacks the specificity required to rule in bacterial infection definitively. While recent studies have suggested NLR cutoffs ranging from 10 to 14 for sepsis prediction,^[7]^ our large-scale cohort indicated a higher optimal cutoff of 30.3 for predicting bacteremia. This further emphasizes that proven invasive infections elicit a significantly more profound hematological stress response than general inflammatory states.

### 4.4. Cost-effectiveness and practicality

Although PCT is widely regarded as a specific marker for bacterial infection, it requires a separate blood draw order and incurs additional costs. In the context of a busy emergency department, the zero-cost nature of automated IG is its greatest strength. It provides a free specificity check derived from the mandatory CBC test. While IG may not replace PCT in complex ICU settings, our findings suggest it serves as an efficient “gatekeeper” in the ED, helping clinicians identify high-risk patients who warrant immediate attention or blood culture collection without waiting for additional test approvals.

### 4.5. Strengths and limitations

Strengths of this study include the large sample size (*N* = 1418), objective microbiological outcome, and the validation of a zero-cost parameter. Limitations include the retrospective single-center design and the modest overall AUCs, underscoring that no single lab value should dictate management alone.

## 5. Conclusion

Automated IG% demonstrates superior specificity for bacteremia compared to WBC and NLR. Integrating IG as a confirmatory marker in a 2-step diagnostic strategy offers a practical, cost-effective method to enhance bacterial infection recognition in the ED. Future prospective, multicenter studies are warranted to validate these findings and further evaluate the clinical impact of incorporating IG% into sepsis diagnostic algorithms.

## Author contributions

**Conceptualization:** Wan-Hua Yang.

**Data curation:** Yi-Ju Yang, Cheng-Pin Huang.

**Formal analysis:** Wan-Hua Yang.

**Methodology:** Wan-Hua Yang.

**Validation:** Wan-Hua Yang.

**Visualization:** Wan-Hua Yang.

**Writing – original draft:** Wan-Hua Yang.

**Writing – review & editing:** Tzeng-Ji Chen.
